# Energy, Waves, and Forces in Bilateral Fracture of the Femoral Necks: Two Case Presentations and Updated Critical Review

**DOI:** 10.3390/diagnostics12112592

**Published:** 2022-10-26

**Authors:** Cosmin Constantin Baciu

**Affiliations:** 1 Department 14 Orthopedy-Traumatology-ATI, University of Medicine and Farmacy Carol Davilla (UMFCD), Dionisie Lupu Street, No. 37, Sector 2, 050474 Bucharest, Romania; cosminbaciu82@gmail.com; 2 Clinical Emergency Hospital (SCUB), Floreasca Route, No. 8, Sector 1, 014461 Bucharest, Romania

**Keywords:** bilateral femoral neck fracture, magnetic resonance imaging (MRI), computer tomograph (CT), X-ray radiographs, ultrasound (US), metabolic disease, shock waves, stress waves, falling dynamics

## Abstract

Two case reports and an updated critical review on bilateral fractures of the femoral neck are presented. Bilateral fractures of the femoral neck have been investigated for at least 80 years and are treated as rare cases. The primary cause is usually considered an external shock; however, aside from high energy shocks (e.g., falling and impact with hard, rigid surfaces, traffic accidents, etc.) the underlying causes of femoral neck frailty have not yet been fully understood. Although not exhaustive, the review spans cases reported as early as 1944 and compares their conclusions in line with medicine developments at the time of the reports until present. The discussion is perhaps controversial at times; it brings to the fore the energy balance between shock waves and stress waves. The two cases reported here add to the review, one highlighting the biomechanics, and the other supporting more recent findings on metabolic disorders, which ultimately lead to enhanced frailty of the femoral neck. Investigation of the fractures has been performed with X-ray radiographs, MRI, and CT, with a follow up using a Doppler US to check blood flow in the lower zone of the limbs. The second case was investigated both for fractures and metabolic diseases, e.g., type I diabetes and kidney failure (dialysis). In Case 1 the second fracture was not observable at the time of admission, and therefore two surgery operations were performed at seven days interval. Taperloc Complete prostheses (Zimmer Biomet) were applied. Case 2 suffered a second fracture in the right hip in the segment above the knee and required better fixation with cables. Despite this, she returned one month later with a new crack in the femur. Case 1 is a typical case of wear consequences on the biomechanics of the hill pad-tibia-femur-femoral neck system, where tension of the neck occurred due to a stress wave rather than a shock wave. This can be proven by the absence of a second fracture from the images first acquired, the only evidence being pain and walking difficulty. Case 2 shows that metabolic diseases can dramatically enhance the frequency of bilateral femoral neck fractures.

## 1. Introduction

Bilateral femoral neck fracture has long been subject to investigation, owing to its rare occurrence and the multiple causes and mechanisms supposedly responsible for its incidence. The primary cause is usually considered external shock; however, aside from high energy shocks (e.g., falling and impact with hard, rigid surfaces, traffic accidents, etc.) the underlying causes of femoral neck frailty, and how the fractures are produced on both sides, have yet to be understood. A study spanning approximately 80 years of investigations does not reveal thousands of cases, and therefore, these types of fractures are valued as rare. It is also difficult to precisely determine whether men or women are more prone to this condition, or if patients of a certain age can be found more frequently in the studies available to date, because data present both sexes and a range of ages, including children. [1,2,3,4,5]. Osteoporosis in older females is claimed as a criterion, but it is not self-consistent [6,7,8,9,10,11,12,13,14,15,16,17].

Underlying conditions, such as bone metabolism disorder, chronic steroid use, renal osteodystrophy, and hypocalcemia, are the main causes of bone frailty, and not of the fracture itself. Convulsions in epileptic patients, or electroshock treatment [18], have resulted in bilateral femoral neck fracture as mentioned in ref. [18], emphasizing the role of muscle contractions. A careful analysis of causes, mechanisms, and effects would allow us to separate the aspects and criteria issued from the problem into (a) underlying causes of bone frailty and (b) mechanisms that have produced bilateral femoral neck fractures. The latter can include high energy trauma [19]. However, a thorough investigation of the possible mechanisms leading to bilateral fracture of the femoral neck should bring to the fore questions related to wave propagation through various segments of the body [18,19,20,21,22,23,24,25,26,27,28].

**Figure 1:**
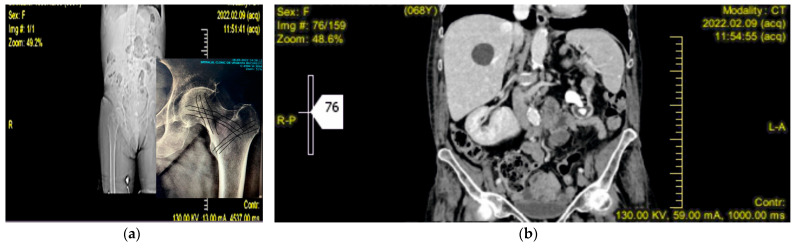
Case 1. (**a**) X-ray radiograph with inset showing the trabeculae of the femoral neck; (**b**) CT before fracture.

**Figure 2:**
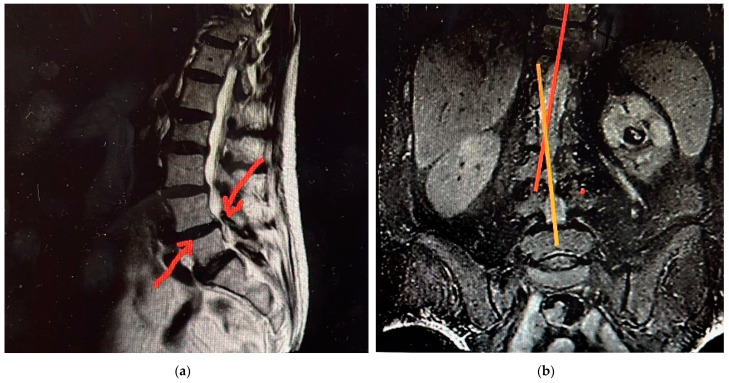
(**a**,**b**) MRI images of the spine for Case 1 following three weeks of sciatica and one week before the bilateral fracture. The inflammation and the discal prolapse associated with lumbar scoliosis remarked could have led to instant pain and caused the fall. The femoral heads are not shown in this image.

In order to understand the mechanisms behind bilateral femoral neck fractures, it is important to first consider the anatomy of the femur. From the internal structures, two trabecular columns (one vertical and one horizontal) are most obvious and are best visualized clinically on antero-posterior radiographs of the hip joint, in which it is seen that they pass deeply through the femoral head, terminating close to the subchondral bone (e.g., Figure 1a). When fine cracks of the femoral neck are produced, they are less likely to be visible on X-ray radiographs; therefore, from the very beginning, an MRI combined with a CT can be more helpful. In this work, Figure 1b shows the CT, and Figure 2a,b, shows the MRI of the preconditions in Case 1. This can lead to an initial misdiagnosis of bilateral femoral neck fracture and to doubtful interpretation of simultaneity in these types of fractures. The horizontal trabeculae, as they traverse the femoral head, are seen to cross the vertical trabeculae at approximately right angles (Figure 2a). The highest stress loads within the femur act on the femoral neck. These are external forces and are enhanced by bone frailty [29,30].

## 2. Case Presentation

**Figure 3:**
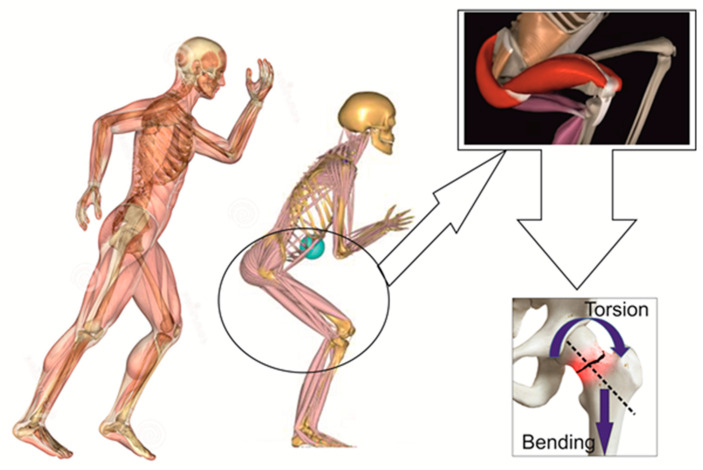
Simulation of the fall that led to the fracture in Case 1.

Case 1. A woman aged 68 was admitted to our clinic with great walking difficulty and strong pain in both hip joints. Her recent history showed three weeks of tough sciatica in the R side and a consequent cessation of any exercise, work outs, and normal walking, thus leading to weakness of the muscles. She had fallen in her flat by stepping on a cylindrical cable and, in an attempt to reduce the height of falling, she performed a forced squat (Figure 3). The X-ray radiographs performed immediately at admission showed a fracture of the R femoral neck, but no sign of cracks in the L side. Additional information from CT, MRI, and US scans [31,32,33] showed coralliform stones in her L kidney, whereas the R side was clean. Ultrasound investigation has shown an incipient degenerative process, but not to such an extent to produce an enhanced frailty of the bones. The US also confirmed, supported by the MRI and CT scans, that the stones in the left kidney were only delaying its normal function and had no major impact on her health. The patient had normal blood pressure, normal blood chemistry values, and a satisfactory general state. The L hip fracture was not observable at the time of admission but only during the surgery on the R hip. The second surgery operation, i.e., on the L hip, was performed after seven days. Non-cement Taperloc Complete prostheses (Zimmer Biomet, Medical Technologies International (MTI), 206 D, 13 September Road, Bucharest, Romania) were applied to each hip. Loading started five days after the second surgery operation. The patient used a walker while assisted by a kinesiotherapist.

Forces acting on the femoral necks are both external and internal. The external forces are generated as a reaction to the ground-hill pad impact and are transmitted through the ankle toward the femoral neck. (Figure 3). Normal movements and body balance are achieved by forces generated by muscles acting on bones. These are known as internal forces. At rest, externally and internally generated forces are in equilibrium. These two kinds of forces yield bending and, respectively, torsion moments upon the femoral neck, with the bending moments being a product of vertical loading during daily activities that create compression at the inferior zone of the femoral neck and tension at the superior one (Figure 3). The vertically oriented trabeculae, located in the inferior zone of the femoral neck, uptake and transfer the vertical load.

Previously published studies show that hip fractures in patients dependent on dialysis are 4.4 times greater than in the general population [34,35], with an incidence of 29.3/1000 people per year [36]. This information relates to unilateral hip fractures, as bilateral ones are still very rare [37].

**Figure 4:**
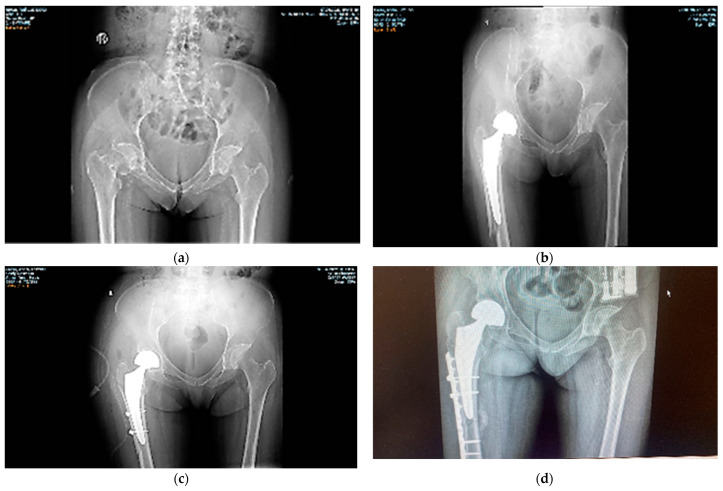
Case 2. (**a**) The bilateral femoral neck fractures are well defined. The patient suffers from type I diabetes and is also dependent on dialysis. (**b**) The R fracture is reduced with a Taperloc prosthesis, but the femur cracked during insertion of the prosthesis and supplementary cables were required to fix the bone (**c**). (**d**) shows the final result, i.e., complete reduction in the fracture in the R femur one month after the first surgery, when the patient returned to the clinic with a new fracture on the same side. The strongest sources for such an enhanced frailty of the bone are presumably her metabolic diseases (see also refs. [5,10,11,12,13,14,20,21,22,23]).

Case 2. A 37-year-old woman, with type I diabetes and dialysis dependency, was admitted to our clinic with a bilateral femoral neck fracture caused by a free-fall domestic accident. The impact with a flat, hard surface had produced the fractures. X-ray radiographs (Figure 4) showed the cracks at the beginning of investigations. Hematology and biochemistry tests were acceptable, considering her complicated health condition and comorbidities. During surgery the patient suffered a second fracture in the right hip in the segment above her knee and required better fixation with cables. She had been cleared for weight bearing after two months. However, she returned only one month later with a new crack in the femur. Surgery was successful (Figure 4d). Loading has been foreseen at the end of the following four months.

In this case, a high energy shock caused the initial fractures, but it must be taken into account that the patient’s bones were dramatically weakened and frail due to diabetes and kidney failure. As one underlying cause of this patient’s bilateral femoral neck fracture, perhaps the major one, we assumed renal osteodystrophy, a consequence of her chronic kidney disease.

External forces generate shock by the waves that propagate from the hill pad/ground interface to the femoral neck, passing through three joints (ankle, knee, and hip), and two big bones (tibia and femur). The femur is the largest bone in the human body.

**Figure 5:**
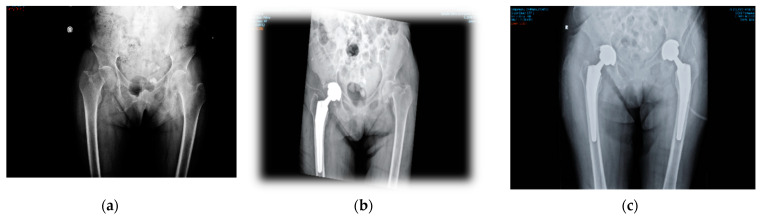
Case 1: X-ray radiographs. (**a**) The R femoral neck fracture is visible initially, but not the L one. (**b**) R femoral neck fracture reduced with prosthesis. The L crack is revealed in surgery. (**c**) The L femoral neck fracture is fixed with prosthesis seven days post R-side surgery.

The highest stress loads within the femur are sustained by the femoral necks. As compared with the reviewed literature on bilateral femoral neck fractures, the two cases reported here show that kidney disfunction/malfunction can influence bone frailty. In Case 1, the fracture is of the stress kind, i.e., caused by repetitive loading of the femoral neck that leads to either compression side (inferior-medial neck) or tension side (superior-lateral neck) stress fractures. In Figure 5, it is shown that the bilateral fracture occurred on the tension side.

In Case 1, a slight weakness of the muscles at walking can be presumed given her history of sciatica on the R side three weeks prior to the fracture. She had fallen following a slip on a cable, which means it was likely a relatively high velocity fall.

It is therefore clear that using appropriate imaging tools, such as CT, MRI, and US, in addition to X-ray radiographs, can support a more precise diagnosis. Furthermore, as thrombosis is known as the main cause of death in hip fractures in general, Doppler US is extremely useful for monitoring blood circulation in the lower part of the limbs.

## 3. Discussion

There are two factors that should be taken into account when analyzing wave propagation in falls: energy (*E*) and momentum (*P*). Therefore, almost all published works on the topic of bilateral femoral neck fractures discuss high energy or low energy impact. At the interface between the human body and the surface of impact, the shock wave and the stress wave act to satisfy energy conservation. During falling, the energy of the body is dominantly kinetic, and it is a function of the body mass and its velocity: E = 0.5m*v^2^. A complete description of energy for a free-falling body should be described by E = m*g*h + 0.5m*v^2^, where *g* is the acceleration of gravity and *h* is the height, from which the free fall happens. Momentum is also a function of mass *m[kg]* and velocity: P = m*v. Force is derived from momentum, F = m*a, where *a* is the acceleration in movement, and is the derivative in time of the velocity. These simple equations are provided to show that forces and velocities play important roles in the mechanics of the human body falling short distances, as domestic accidents often lead to bilateral femoral neck fractures. This approach was adopted in Case 2. It has been shown by other authors [37] that when v > 3 m/s the probability of a bilateral femoral neck fracture occurring is the highest. Domestic accidents can be caused by various factors, such as mistaken shifting of weight, slipping, etc. The velocity in the latter is higher than in the former [37], and in agreement with the energy and momentum conservation highlighted above, body mass plays an important role. Thus, the higher the initial position, the more energetic the impact. Therefore, attempts to reduce the height of falling (e.g., from standing to kneeling) would diminish the energy dissipation at the impact point. This is the approach that matches Case 1 (see also Figure 3). The entire dissipated energy is of the vibration kind, i.e., we start talking about waves and their propagation to determine whether it is a question of shock waves or stress waves [37,38,39,40].

An updated review on literature concerning bilateral femoral neck fractures would begin with the reports of H.D W. Powell [18], which present a collection of cases (see Tables I–III in (ref. [18], pp. 238–243)), most of them being patients in a psychiatric hospital. Table I ([18], pp. 238–240) shows listed cases that have not previously been reported. Most of these patients had been treated with electroconvulsive therapy, leading to strong contractions causing fracturing of femoral necks and even thoracal vertebrae. There is no question of falling, and the entire dissipating energy belongs to the stress waves in response to the shock waves induced by the therapy, leading to contractions. Statistics on sex and age show male predominance (12 men and eight women), aged between 26 and 71. It is difficult, then, to claim that women and older individuals were more exposed to bilateral femoral neck fractures. However, the collection of cases in Table I ([18], pp. 238–240) touches quite restrictively on the subject as all patients had been diagnosed with psychiatric conditions, and had suffered long stays in hospital. There was no mention of malignancy, osteodystrophy, or other metabolic conditions that could have influenced the frailty of bones. Table II ([18], pp. 241–242) summarizes cases of accidents reported by other authors. It is notable that renal abscesses, pulmonary tuberculosis, diabetes, and severe rheumatoid arthritis are mentioned in connection with diseased femoral necks, but without detail. Table III ([18], p. 243) contains cases of therapeutically induced convulsions, with predominance in male patients.

The comprehensive work published by H.D.W. Powell in 1960 was focused on femoral neck fractures occurring mainly through contractions following electroconvulsive therapy. Hormone activity [24,41], metabolic diseases affecting the bones, and deficiencies in vitamin D or calcium [20,42], were not investigated. Since the 1960s, medical research has evolved tremendously within all branches, including in orthopedics, traumatology, metabolic diseases, and others. Though some authors tend to claim that older females have a predisposition to bilateral femoral neck fractures, recent case reports show that children with a vitamin D deficiency [1,2,3], were subject to this kind of fracture, as well as a young male who had been deprived of sunlight for several years [42]. Atraumatic bilateral fracture of the femoral neck in young male patients with suspected osteomalacia [4] and low-velocity simultaneous bilateral femoral neck fracture following long-term antiepileptic therapy [5,6] have also been reported recently, but are very rare indeed.

Another underlying cause of bone fragility causing bilateral femoral neck fracture is osteoporosis, encountered both in young, pregnant female patients, [5,7,8,9,10,11,12,13,14,38], young adult females with amenorrhea [24], or postpartum [41], and also in aged patients, both women and men [16,17,43,44,45]. Supposedly, metastatic conditions, as in prostate carcinoma described in ref. [45], can lead to osteoporotic fractures in men, although they are three times less prone to osteoporotic fractures than women [45,46]. Hormone suppression following, corticosteroid treatments, for example, can lead to loss of bone density in men. Among the metabolic conditions found in patients with bilateral femoral neck fractures, the works reviewed in this study mostly emphasize hypocalcemia [21,22,23,25] and renal failure, with dialysis where it was the case [22,34,35,36,37]. Case 2, which we presented here, adds to this category of patients.

## 4. Conclusions

An attempt to analyze the causes, mechanisms, and effects of bilateral femoral neck fractures has been made in this study. The approach considers energy, waves, and forces that may lead to the assumption that shock waves are “the action upon the bone structure” and stress waves are its “reaction”, in terms of Newton’s laws.

This work includes two recent case presentations within the frame of an updated critical review. It allows us to separate the aspects and criteria of the problem into (a) underlying causes of bone fragility and (b) mechanisms that produce bilateral femoral neck fractures.

Case 1 is a typical case of war consequences on the biomechanics of the hill pad-tibia-femur-femoral neck system, where compression of the neck occurred due to a stress wave rather than a shock wave. This can be proven by the absence of a second fracture from the images first acquired, the only evidence being pain and walking difficulty. Case 2 shows that metabolic diseases can dramatically enhance the frequency of bilateral femoral neck fractures. Case 1 adds to the deductions of the first comprehensive case report in the 1960s, but without supporting the conclusion that men are more prone to stress wave-induced bilateral neck fractures than women, owing to their long, lean muscles. Still, bilateral fractures of the femoral neck are rare and these factors remain difficult to assess, as they have previously occurred at the same intensity at the same time. Fine cracks can grow and become visible after a period of time. Additional analyses, e.g., CT, MRI, and US scans, in support of X-ray radiographs, are extremely useful for a more precise early diagnosis, as well as for understanding pre-existing conditions related to a patient’s history. Although Case 1 has stones in her L kidney, as shown by US, MRI, and CT scans, this cannot be considered a comorbidity; as a metabolic disease, no evidence of malfunction arose from the biochemistry except from a slight delay in excretion from the L kidney. Therefore, the only common feature of the two cases is the bilateral fracture of the neck of the femur caused by falling of some kind.

“Spontaneous” bilateral femoral neck fracture is not the most suitable term, because it is even not clear when “simultaneous” is entirely appropriate.

Based on the review, a standard treatment protocol should comprise of:

(a) a clinical and paraclinical examination; history of the fracture as told and remembered by the patient; history of comorbidities and their respective treatments; and continuity of medication in cases such as diabetes, dialysis, heart disease, and others of the chronic kind.
(b) imaging and spectral techniques in support of primary X-ray radiographs, to get a more precise picture of the case and diagnostic.
(c) surgery and appropriate manipulation and fixation of the bones, using the most suitable devices (screws, cables, cement/non-cement prostheses, etc.).
(d) post-surgery care, according to the evolution of the patient.

The literature review is not exhaustive, because there might be cases not yet or not at all reported. The two cases we have presented here are very recent and add to the reviewed literature.

## Data Availability

Not applicable.
